# Quantifying the ozone and ultraviolet benefits already achieved by the Montreal Protocol

**DOI:** 10.1038/ncomms8233

**Published:** 2015-05-26

**Authors:** M. P. Chipperfield, S. S. Dhomse, W. Feng, R. L. McKenzie, G.J.M. Velders, J. A. Pyle

**Affiliations:** 1 School of Earth and Environment, University of Leeds, Leeds LS2 9JT, UK; 2 National Centre for Earth Observation (NCEO), University of Leeds, Leeds LS2 9JT, UK; 3 National Centre for Atmospheric Science (NCAS), UK; 4 National Institute of Water and Atmospheric Research (NIWA), Lauder Private Bag 50061, New Zealand; 5 National Institute for Public Health and the Environment, PO Box 1, Bilthoven 3720 BA, The Netherlands; 6 Department of Chemistry, University of Cambridge, Cambridge CB2 1EW, UK

## Abstract

Chlorine- and bromine-containing ozone-depleting substances (ODSs) are controlled by the 1987 Montreal Protocol. In consequence, atmospheric equivalent chlorine peaked in 1993 and has been declining slowly since then. Consistent with this, models project a gradual increase in stratospheric ozone with the Antarctic ozone hole expected to disappear by ∼2050. However, we show that by 2013 the Montreal Protocol had already achieved significant benefits for the ozone layer. Using a 3D atmospheric chemistry transport model, we demonstrate that much larger ozone depletion than observed has been avoided by the protocol, with beneficial impacts on surface ultraviolet. A deep Arctic ozone hole, with column values <120 DU, would have occurred given meteorological conditions in 2011. The Antarctic ozone hole would have grown in size by 40% by 2013, with enhanced loss at subpolar latitudes. The decline over northern hemisphere middle latitudes would have continued, more than doubling to ∼15% by 2013.

## Abstract (web summary)

Ozone-depleting substances have been controlled by the 1987 Montreal Protocol, ensuring atmospheric concentrations are now in decline. Here, the authors use a 3D model and suggest that these controls have already had significant benefits, with much larger ozone depletion than previously thought avoided by the protocol.

## Graphical abstract

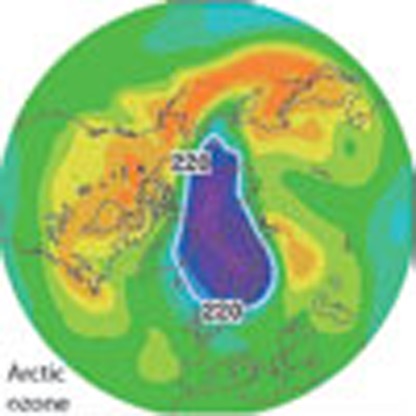

Concern over the depletion of the ozone layer started in the 1970s with the suggestion that chlorine from chlorofluorocarbons could reach the stratosphere and cause ozone loss^1^. Research activities intensified greatly in the mid-1980s following the surprise discovery of significant ozone depletion over the Antarctic^2^, the so-called ozone hole. This large decrease in ozone was explained by chlorine- and bromine-catalysed loss acting in the particularly cold conditions of the Antarctic polar lower stratosphere, which allow polar stratospheric clouds to form (see, for example, Solomon^3^). Ozone depletion was subsequently observed in the Arctic springtime stratosphere, but the loss was much smaller than the Antarctic and more variable from year to year^4^,^5^. Cold years in the Arctic stratosphere, such as 1994/95, lead to an integrated column loss of around 120–140 DU (∼35%), while warm years, without polar stratospheric cloud occurrence, produce essentially zero loss.

The discovery of the Antarctic ozone hole helped stimulate the initial signing in 1987 of the Montreal Protocol, an international treaty to limit production of chlorine- and bromine-containing ozone-depleting substances (ODSs). The Montreal Protocol has since been strengthened greatly through subsequent amendments and adjustments, supported by ongoing research, which has enhanced our understanding that ozone loss, in the polar regions and globally, is driven by chemical processes involving chlorine- and bromine-containing gases, arising mainly as breakdown products of ODSs. With global compliance with Montreal Protocol regulations, atmospheric chlorine peaked at ∼3.6 parts per billion by volume (p.p.b.v.) in 1993 at the surface and a few years later in the stratosphere, and then began to decline. The current tropospheric loading is 10% below the 1993 peak value.

Most of the major ODSs have long lifetimes (many decades) in the atmosphere^6^, so a significant time delay is expected before the reduction in atmospheric emissions of chlorine and bromine translate into an increase in stratospheric ozone. Column ozone amounts in the northern middle latitudes reached their minimum values in the mid-1990s at about 6–8% below the 1964–1980 mean^7^, due to the combination of elevated stratospheric chlorine and bromine with enhanced aerosol loading after the 1991 eruption of Mt Pinatubo. Mean northern mid-latitude ozone values at the present day are still around 4% below the long-term mean^8^. The Antarctic ozone hole continues to re-appear each spring; stratospheric chlorine is not expected to return to 1980 levels, when the Antarctic ozone hole was first detectable, until about 2050. Substantial Arctic ozone loss also continues to be observed in the late winter/early spring of some years when polar stratospheric temperatures are particularly low. The largest Arctic ozone loss observed to date occurred in the recent cold winter of 2010/11 (ref. 9). By early April 2011, about 75% of the ozone had been destroyed in a limited altitude region of the polar lower stratosphere. Despite this relatively large local loss there were substantial differences in the spatial extent and the degree of local depletion between a typical Antarctic ozone hole and the 2011 Arctic loss. Near complete removal of ozone occurs annually in the Antarctic lower stratosphere while the maximum loss in the 2011 in Arctic is estimated at just over 80% at 18–20 km within a polar vortex covering a much smaller area than in the south. By late March, around 45% of the Arctic polar vortex had column ozone values below 275 DU. However, the observed column did not fall below the threshold for the Antarctic ozone hole, taken as 220 DU. Therefore, by that definition an ‘Arctic ozone hole' did not occur.

Several studies have investigated the benefits of the Montreal Protocol. Prather *et al.*^10^ first discussed the possible impacts if the protocol had not come into force. Later studies have attempted to use atmospheric models to quantify the benefits. These have focused on the avoided future impacts later this century, assuming a continued strong growth in ODS emissions, and stratospheric chlorine. Morgenstern *et al.*^11^ used a coupled chemistry-climate model (CCM) to compare the atmosphere in 2030 with and without the effect of the Montreal Protocol. By 2030 their assumed stratospheric chlorine loading was 9 p.p.b.v. In addition to increased stratospheric ozone loss, they found a feedback on stratospheric dynamics and an impact on surface climate. Newman *et al.*^12^ used a similar CCM to investigate the impact on an uncontrolled 3% per year growth in chlorine through 2065. As expected, they found very large stratospheric ozone depletion and also large temperature changes in response to the ozone loss. Garcia *et al.*^13^ used their CCM to study the chemical processes responsible for this possible collapse of the ozone layer in the mid twenty-first century. Finally, Egorova *et al.*^14^ also used a CCM to study the impact of the Montreal Protocol on the ozone layer and again focused mainly on the large losses that would have occurred by the end of this century. Their analysis did extend from 1987 to 2100 but, as they were using a free-running CCM like all of the above studies, internal model variability prevented them from being able to diagnose the impact of the Montreal Protocol during specific years of the recent past.

These ‘World Avoided' studies have thus mainly looked far into the future under the assumption that no action would have been taken to control ODS emissions, and compared the results with those expected with full implementation of the Montreal Protocol. In the face of the expected mounting evidence of an impact it seems quite unlikely that ODS emissions would have continued to grow at the assumed uncontrolled rates without some policy action by the middle of this century. Furthermore, these studies have partly focused on the coupled climate impact of large O_3_ loss, but climate models often have temperature biases that make accurate quantitative modelling of the strongly temperature-dependent polar ozone loss extremely challenging.

In contrast, off-line chemical transport models (CTMs) have been shown^15^,^16^,^17^ to give an accurate simulation of stratospheric ozone loss when they use meteorological analyses—our best estimate of the ‘real' meteorology. CTMs have also been developed with a focus on accurate and complete representations of chemical processes, although CCMs often use computationally fast schemes in more complex climate models. Therefore, a good CTM is likely to produce a more faithful simulation of the present and past atmosphere than a good CCM.

Here we use CTM calculations, covering the past two decades during which the growth of atmospheric chlorine and bromine abundances first slowed and then started to decline, to assess the benefits already achieved by the Montreal Protocol. We show that much larger ozone depletion than observed has been avoided by the protocol. A deep Arctic ozone hole, with column values <120 DU, would have occurred given the meteorological conditions in 2011. The Antarctic ozone hole would have grown in size by 40% by 2013, with enhanced loss at subpolar latitudes. The decline over northern hemisphere middle latitudes would have continued, more than doubling to ∼15% by 2013. This ozone loss would have led to increases in surface ultraviolet of up to 8–12% in Australia and New Zealand and 14% in the United Kingdom, and consequent increases in skin cancer.

## Results

### Global ozone

**Figure 1:**
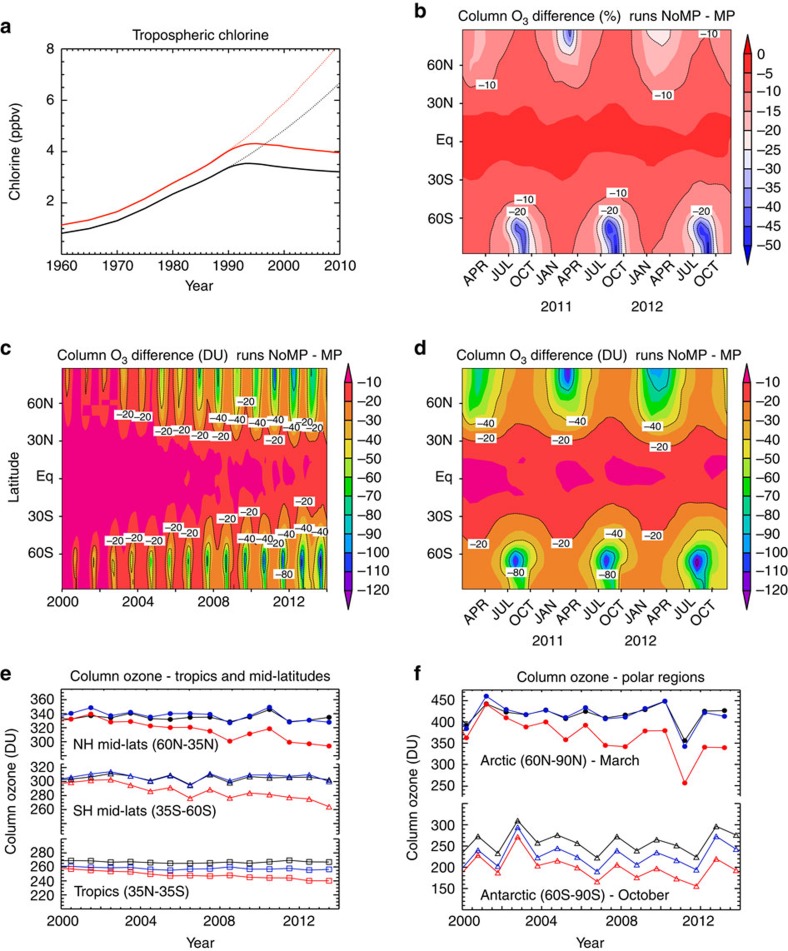
Calculated impact of Montreal Protocol on atmospheric halogens and column ozone. (**a**) Time series of surface chlorine (black) and surface equivalent chlorine (chlorine+50 × bromine, red) from observations (solid lines) and assumed 3% growth (dotted lines). (**b**–**d**) The difference in zonal mean monthly mean column ozone (DU) between run MP and run NoMP for (**b**) 2010–2012 (%), (**c**) 2000–2013 (DU) and (**d**) 2010–2012 (DU). (**d**) The same results as **c** on an expanded time axis for clarity. (**b**) The same results as **d** but as a percentage change. (**e**, **f**) Comparisons of SBUV observed column ozone^37^ in different latitude regions (black) with model runs MP (blue) and NoMP (red).

Figure 1 shows the difference between the modelled evolution of column ozone since the year 2000 between the ‘MP' model run, which includes the effects of the Montreal Protocol and is based on observed atmospheric ODS loading, and the ‘NoMP' model run, which assumes continued growth of ODSs production at 3% per year after 1987 (ref. 18; Fig. 1a, plotted until 2010 which corresponds to the stratospheric loading until about 2014). MP is our best reconstruction of the recent evolution of the ozone layer, and is in excellent agreement with observations: the model simulates the global column ozone distribution^19^ to within a few DU in mid-latitudes and the Arctic spring and to within 10 DU in the tropics (Fig. 1e,f). Meanwhile, the NoMP run projects what could have occurred given our specified ODS growth rate without the Montreal Protocol: it describes the ‘World Avoided'.

There are significant differences between the two runs, which become greater with time. These differences are most evident in the polar regions of both hemispheres. Figure 1 also shows that a decrease of around 10 DU (∼5%) has been avoided by the Montreal Protocol in the tropics, where observed ozone trends have been small. The larger differences over populated middle latitudes indicate that the Montreal Protocol has already had a very significant effect in protecting the ozone layer there. Without the Protocol ozone columns over northern middle latitudes would by 2010 have been between 5 and 10% lower than has occurred with the Protocol. Recall that the observed ozone, in response to the Protocol, is still about 4% lower in northern middle latitudes than the 1964–1980 long-term mean. So, the Protocol has by 2014 already saved us from at least a doubling in ozone depletion.

**Figure 2:**
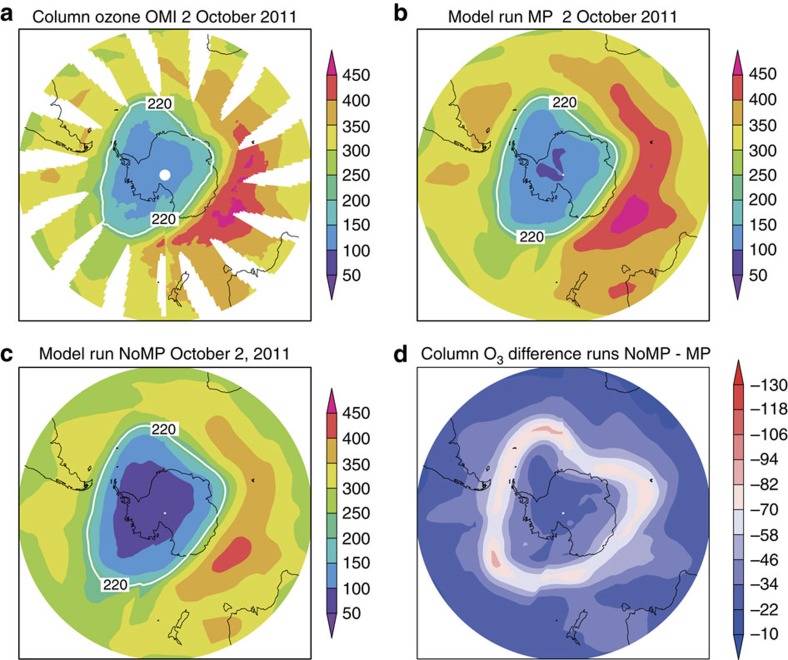
Column ozone in the Antarctic from satellite observations and model for October 2011. Column ozone (DU) on 2 October 2011 (**a**) observed by OMI, (**b**) from model run MP and (**c**) from model run NoMP (with the 220 DU contour indicated in white). (**d**) Difference in column ozone between runs NoMP and MP.

The most dramatic measure of ozone loss is the Antarctic ozone hole. Figure 1 shows that without the Montreal Protocol ozone loss at both poles would have become very much larger. In the Antarctic, the observed ozone loss is already essentially 100% in the lower stratosphere by early October. Therefore, the extent of ozone depletion can only be increased by loss at higher/lower altitudes or at lower latitudes, or lasting for a longer period. Figure 2 shows a comparison of column ozone in October 2011 from runs MP and noMP. Without the Montreal Protocol our model shows around 100 DU additional loss in a substantial edge region of the polar vortex in spring by 2014 (Figs 1d and 2d).

### Arctic ozone hole in 2011

**Figure 3:**
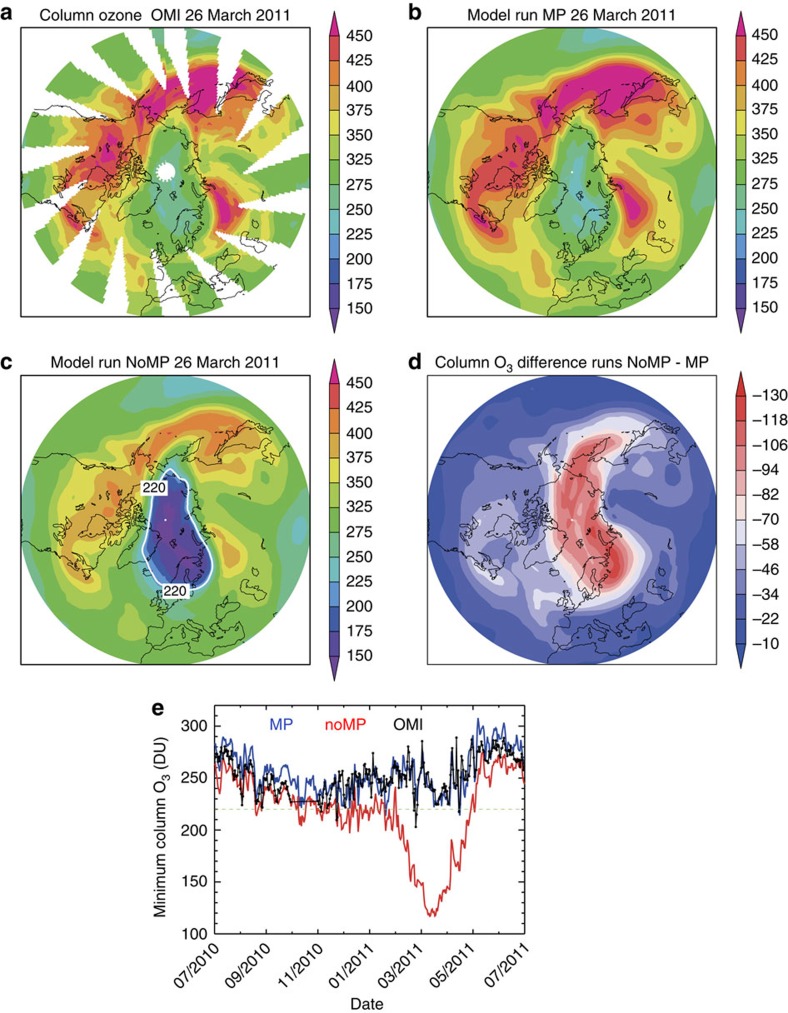
Evolution of column ozone in the Arctic from satellite observations and model for winter 2010/11. Column ozone (DU) on 26 March 2011 (**a**) observed by OMI, (**b**) from model run MP and (**c**) from model run NoMP (with the 220 DU contour indicated in white). (**d**) Difference in column ozone between runs NoMP and MP. (**e**) The daily minimum O_3_ column in the Arctic region (latitude >45°N) from mid-2010 to mid-2011 as observed by OMI (black points), along with equivalent model results from run MP (blue) and run NoMP (red).

We consider next a particularly dramatic polar feature, the very large additional Arctic ozone loss, which was avoided by the Montreal Protocol in 2011. Figure 3e shows the time series of minimum column ozone in the Arctic in winter 2010/11 observed by the Ozone Monitoring Instrument (OMI). The observed column has a local minimum of around 230 DU, lasting several weeks, during the main phase of chemical ozone depletion in March. Results from the run MP show excellent agreement with OMI, further demonstrating the ability of the TOMCAT CTM to reproduce the observed evolution of the ozone layer when forced by observed ODS boundary conditions and appropriate meteorology. In strong contrast to run MP and the observations, the NoMP integration indicates a minimum column below 120 DU, a value that is quite unprecedented in the observational record. Large depletion also lasts not weeks, but months. These results are further illustrated in Fig. 3a–d, which show maps of observed and modelled column ozone on 26 March 2011. The observed region of relatively low column ozone (250–275 DU) is clearly visible and is well captured by model run MP. With the increased chlorine and bromine in run NoMP there is a further dramatic decrease in column ozone by up to 130 DU over a wide region of the Arctic. The 220 DU contour in Fig. 3c clearly demonstrates that without the Montreal Protocol a deep Arctic ozone hole would have occurred in 2011.

### Recent decadal variations in Antarctic and Arctic

**Figure 4:**
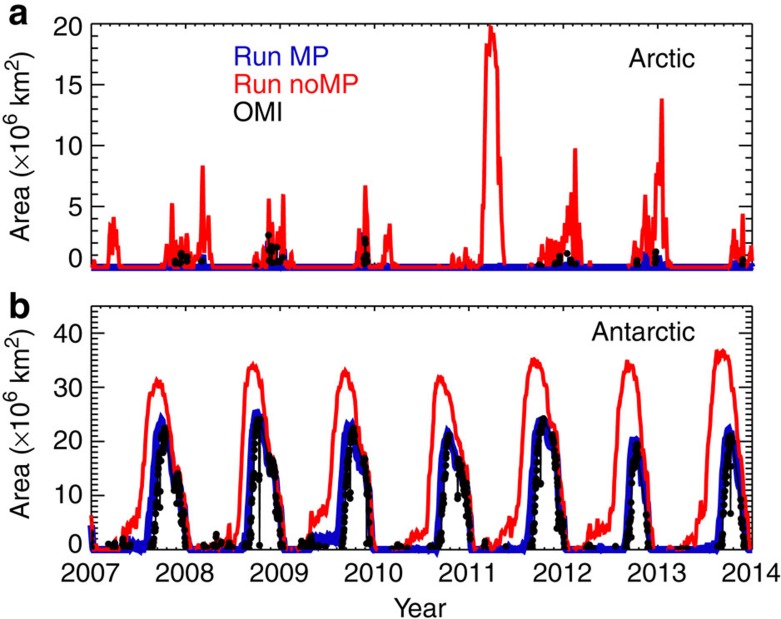
Area extent of the Antarctic and Arctic ozone holes. The area of the ozone hole is defined as the area with column ozone<220 DU. (**b**) The observed area of the Antarctic ozone hole from 2007 to 2014 estimated from OMI data (black symbols). (**a**) For the Arctic, the observed ozone hole area is near zero in all years. The figure also includes the ozone hole areas calculated from model runs MP (blue) and NoMP (red).

Although the Arctic 2010/11 stands out as a major signature of additional polar ozone loss, with around 110 DU lower ozone in the monthly mean zonal mean, Fig. 1 shows that the Montreal Protocol has already had an impact in other (warmer) years in the Arctic, avoiding much larger ozone loss than was actually observed. The impact of the Montreal Protocol in recent years on the size of the Antarctic and possible Arctic ozone holes is shown in Fig. 4. In the Antarctic, the MP integration shows excellent agreement with the size of the ozone hole derived from OMI with, for example, a peak area of 25 × 10^6^ km^2^ in 2008 and some small interannual variability. Without the Montreal Protocol, the size of the hole would have already increased substantially, by ∼40% to over 37 × 10^6^ km^2^ in 2013. Moreover, the onset of the low (<220 DU) columns would start much earlier in the season. Neither the observations nor model run MP show the occurrence of an Arctic ozone hole. As noted previously, although there was considerable chemical destruction of ozone in 2011 (ref. 9), this was offset by dynamical effects that mitigated the ozone depletion. These effects are well captured in our model. However, as discussed above, an Arctic ozone hole would clearly have occurred under the meteorological conditions of 2010/11 without the Montreal Protocol. It is also worth noting the large size of the calculated Arctic ozone hole in 2011 in run NoMP. With an area near 20 × 10^6^ km^2^, the Arctic ozone hole would have been a similar size to the actual Antarctic hole observed in the 1990s and 2000s (see also the region contained by the 220 DU contour in Fig. 3c). Figure 4 also shows that many other recent winters (for example, 2012/13) would also have experienced periods with minimum column ozone below 220 DU, that is, the model run indicates that smaller Arctic ozone holes would have become a regular occurrence.

## Discussion

Ozone absorbs radiation in the ultraviolet and infrared regions of the electromagnetic spectrum. As a result, ozone depletion affects the temperature structure of the atmosphere, which has important long-term consequences for circulation and climate. In addition, ozone depletion increases solar radiation at the surface thereby increasing the risk of damage to humans, flora and fauna. For example, model studies have linked the depletion of O_3_ in the Antarctic to a delay in the breakdown of the polar vortex through decreased heating^7^,^8^,^20^,^21^. Without the Montreal Protocol, the enhanced ozone loss would have increased this effect and likely led to a further extension of the duration of the Antarctic vortex. Antarctic ozone depletion has also been linked to an observed change in the surface climate at high southern latitudes. The depletion has led to a change in the Southern Annular Mode with a poleward shift in the position of the summertime southern hemisphere jet^22^,^23^. Changes in high-latitude surface temperatures and in southern hemisphere rainfall have also been attributed to the ozone loss^24^,^25^. We do not discuss these in detail here, and these effects cannot be modelled in our offline CTM, but we simply point out that even greater loss than observed would certainly have had further important consequences.

Stratospheric ozone depletion leads to an increase in potentially harmful radiation at the surface, assuming that other absorbers, including clouds and aerosol, remain the same. Previous ‘World Avoided' studies have assessed the impact of changes in surface ultraviolet into the future^26^. Based on the Morgenstern *et al.* study^11^, Van Dijk *et al.*^27^ estimated the number of cases of skin cancer avoided by 2030 at very roughly 2 million, or about 14% fewer skin-cancer cases per year. The much more substantial ozone declines in 2065 predicted in the study by Newman *et al.*^12^ would presumably have led to very much higher skin cancer rates by 2100 (ref. 28). Many other factors can affect ultraviolet penetration to the surface and assessing the knock-on health impacts is very uncertain^29^. Nevertheless, the world avoided, with large additional ozone loss, would necessarily have seen important increases in surface radiation.

**Figure 5:**
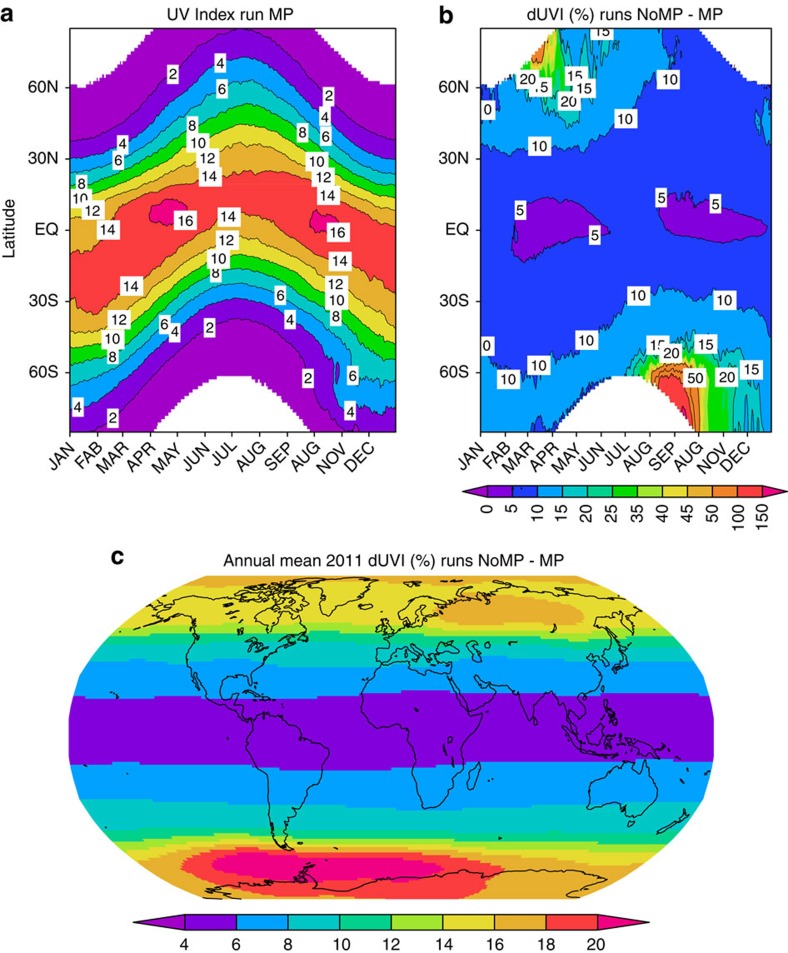
The impact of the Montreal Protocol on surface ultraviolet index for 2011. (**a**) The daily zonal mean sea-level ultraviolet index at local noon calculated from the column ozone from run MP and a radiative transfer model^39^. (**b**) The percentage difference in daily zonal mean ultraviolet index at local noon between model run NoMP and run MP. (**c**) The percentage difference in annual mean ultraviolet index at local noon between model run NoMP and run MP.

We can assess the changes already avoided using our realistic calculations of ozone to estimate the ultraviolet index (UVI). The UVI describes the level of solar ultraviolet radiation at the Earth's surface, ranging from zero upward—the higher the index value, the greater the potential for damage to the skin and eye, and the less time it takes for harm to occur^30^. Figure 5a shows the noontime sea-level UVI calculated using the daily column ozone for 2011 from model run MP, showing a strong gradient between the tropics and high latitudes. The additional ozone depletion in run NoMP causes an increase in UVI at all latitudes (Fig. 5b). The largest changes occur at the edge of the Antarctic vortex in September–November, with an increase of 20–100%. Following the large ozone loss in the Arctic in 2011, the UVI increases by a mean of over 15% at 60°N in March. In the tropics, the UVI increases by about 5% all year round.

Figure 5c shows the percentage change in the 2011 annual mean noon UVI between runs NoMP and MP. This is a measure of the change in the sun-burning dose of ultraviolet. Without the Montreal Protocol, these ultraviolet changes would have been a strong function of latitude, ranging from more than a 20% increase in parts of Antarctica, to 5% or less in the tropics. At mid-latitudes, where skin types of the resident populations are typically more sensitive to ultraviolet damage, the percentage changes are potentially important. For example, in the most populated areas of Australia and New Zealand, which currently have the highest mortality rates from skin cancer^31^, increases would have been 8–12%; and in Northern Europe, including the United Kingdom, increases would have exceeded 14%. Health impacts of these changes in UVI are complex and difficult to quantify. However, changes as large as these would have had potentially serious consequences in the decades that followed. In the absence of any changes in sun-exposure patterns, a 5% increase in sunburning ultraviolet is expected to lead to larger increases in the two most common forms of skin cancer: about 15% and 8% in incidence rates for squamous and basal cell carcinoma, respectively^32^. Effects on melanoma rates, with its higher mortality, are less certain because the action spectrum for melanoma in humans has not been measured. More quantitative statements would require analyses of the type described by van Dijk *et al.*^27^, but focusing on a later period to take account of the large time lag between ultraviolet exposure and the development of skin cancer.

The Montreal Protocol is rightly seen as a seminal international agreement, which is successfully protecting the ozone layer. In this paper, we have shown that, just over two decades since it was ratified, the Montreal has already had major beneficial impacts, including avoiding an Arctic ozone hole.

## Methods

### Model configuration and experimental design

We have used the TOMCAT/SLIMCAT off-line three-dimensional (3D) CTM to calculate the impact of the Montreal Protocol on stratospheric ozone^33^. The model has been widely used in previous studies of stratospheric chemistry^34^ and reproduces stratospheric ozone well. The model includes a detailed treatment of stratospheric chemistry, including a full description of processes related to polar ozone depletion. The model is forced by ERA-Interim analyses provided by the European Centre for Medium-Range Weather Forecasts^35^. The model was integrated in two experiments at a horizontal resolution of 2.8° × 2.8° with 32 levels from the surface to ∼60 km. The control run (MP) was forced by observed surface mixing ratios of source gases from 1979 to 2013. Run NoMP was identical to run MP but used an alternative scenario for chlorine- and bromine-containing source gases after 1987. This scenario assumes a 3% per year growth rate in the production of ODSs.

We drive the world avoided model simulation with meteorological analyses for the period between 1979 and 2013 but allow the ODS concentrations to grow, unaffected by the Montreal Protocol. Ozone is a climate gas so, had there been no Protocol, stratospheric temperatures would have slowly evolved to a different mean state, with a consequent feedback on dynamics. The most obvious observed effect of ozone depletion has been a cooling and strengthening of the Antarctic polar vortex^20^,^21^, which itself would exert a feedback tending to deepen and prolong the ozone hole, with subsequent impacts on tropospheric climate. We believe that our neglect of this feedback during the short time period since the implementation of the Montreal Protocol in our ‘World Avoided' scenario is a justifiable approximation globally, compared with using the meteorology simulated in a climate model. However, the approximation is likely to lead to a small underestimate of the avoided ozone depletion in the Antarctic.

### Satellite data

To compare to our MP model simulation, we use observations from the OMI^36^ level 3 (OMTO3d) total column data. The OMTO3d are TOMS-like (Total Ozone Mapping Spectrometer) daily gridded datafiles, generated by gridding and merging only high-quality level 2 measurements (based on TOMS-like algorithm) for a given day. Data are available from 1 October 2004 at 1.0° × 1.0° resolution and is obtained from http://disc.sci.gsfc.nasa.gov/Aura/data-holdings/OMI/omto3d_v003.shtml. As OMI data are only available from 2004, Figure 1e and f shows merged Solar Backscattered Ultraviolet (SBUV) ozone data (v8.6). These are monthly mean zonal and gridded average products constructed by merging individual SBUV/SBUV/2 (total and profile ozone) satellite data sets^37^ (see http://acd-ext.gsfc.nasa.gov/Data_services/merged/).

### Ultraviolet Index

Skin-damaging ultraviolet radiation is usually taken to be the spectral ultraviolet irradiance weighted by the erythemal action spectrum^38^. This erythemally weighted ultraviolet (UVery) is usually measured in Wm^−2^. However, for public dissemination, the UVI is widely used, where UVI=40 × UVery. UVI values were pre-calculated and tabulated as a function of ozone and solar zenith angle. The calculations were carried out with the tropospheric ultraviolet and visible (TUV) radiative transfer model^39^, using the 8-stream pseudo-spherical discrete ordinate method, and apply to snow-free surfaces near sea-level under cloudless and unpolluted skies. Corrections have been applied to take account of seasonal changes in Sun–Earth separation. However, absolute values should be treated with caution, especially in polluted sites, because UVI is sensitive to factors other than ozone. For example, UVI increases by at least 5% per kilometre of altitude, and can increase by more than 20% when surfaces are snow-covered. On the other hand, extinctions by aerosols in polluted locations can lead to substantial reductions in UVI. In principle, corrections can be applied to take account of such differences. However, as we are primarily interested in differences between two scenarios for which those factors are the same, these have not been applied.

## Additional information

**How to cite this article:** Chipperfield, M. P. *et al.* Quantifying the ozone and ultraviolet benefits already achieved by the Montreal Protocol. *Nat. Commun.* 6:7233 doi: 10.1038/ncomms8233 (2015).
